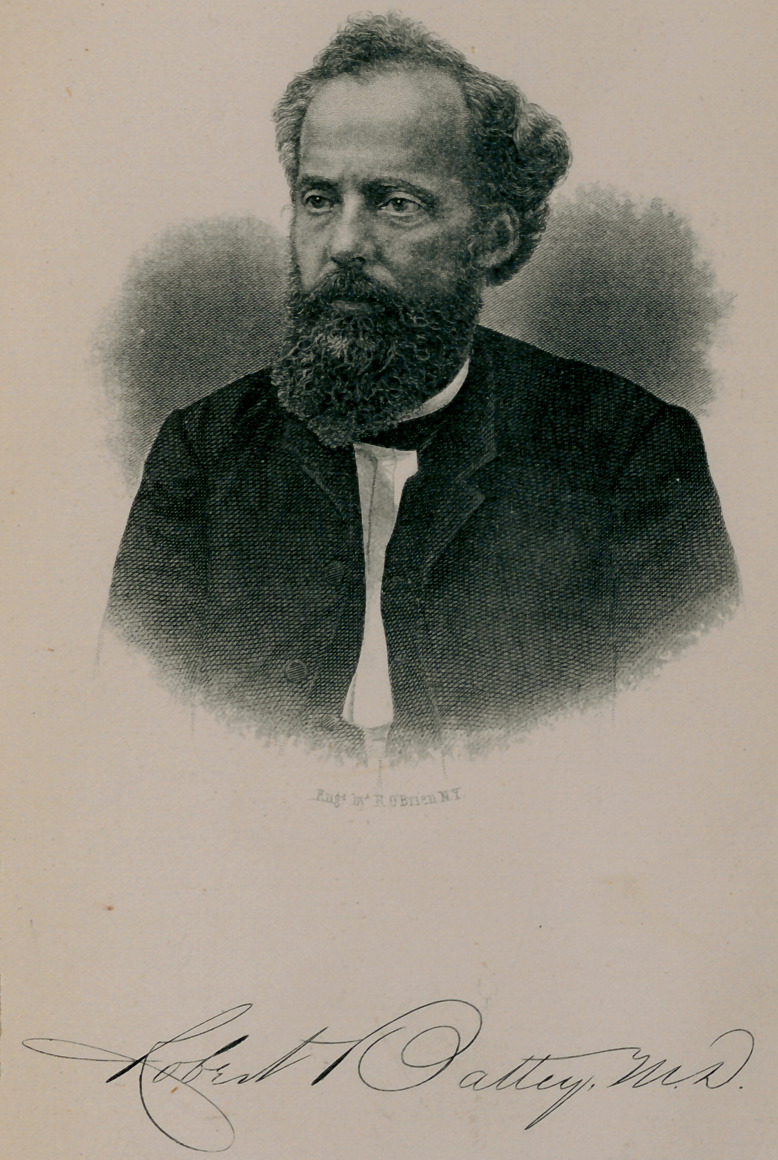# Our Portrait Gallery

**Published:** 1884-11

**Authors:** 


### OUR PORTRAIT GALLARY.

### ROBERT BATTEY, M. D.

There are comparatively few persons who make their lives truly successful, and establish reputations which live after they are dead in the history of achievements for the good of mankind. True, there are multitudes who amass wealth, or who win perishable renown; but such as accomplish solid, enduring benefits for our race are as seldom met as oases in the arid desert wastes. Those who delve into the mysteries of science and develop truths unknown before, are always contributors to the happiness of humanity, and win for themselves honors that are imperishable. To this latter class belongs

DOCTOR ROBERT BATTEY, OF ROME.

Dr. Battey is a native Georgian. He was born in the city of Augusta on the 26th day of November, 1828. His parents, Cephas and Mary Magruder Battey, died when he was only eleven years of age. Left thus early to battle with the adverse circumstances of life, his triumphs are for this reason the more to be appreciated and admired. He received a partial literary education at an academy in Amherst, Massachusetts, devoting himself at his first opportunity to the study of chemistry and pharmacy, which were regarded, almost intuitively, as the foundation of his future labors and success. But his financial condition required him to forego for a time the pursuit of that knowledge which was in accord with his natural aspirations, and he engaged as a dry-goods clerk in the store of Zach Chandler, in Detroit, Michigan—the identical man who in after

Eng^d by R. O'Brien N.Y.

Robert Battey, M.D.

years acquired such fame as a politician. Even while thus employed he devoted every hour of leisure to his favorite study, laying a solid basis for a life of usefulness.

In 1847 he fortunately secured employment in a drug house at Marshall, Michigan, where he was afforded opportunity for a practical use of much that he had learned from text-books in chemistry and pharmacy. The following year he determined to return to his native State, and succeeded in securing a favorable situation as clerk in a drug-store at Rome, Georgia. In 1849 he began the drug business at that place on his own account, and entered at once on a career of experiment and study, by which he was soon distinguished as an enterprising and accomplished chemist and pharmacist, commanding at the same time a large and profitable trade.

With limited capital he was under the necessity of personal labor in his own establishment, and this fact no doubt contributed the more to his discoveries. He was devoted to his chosen profession, and freely expended money, time and talents in efforts to make it beneficial to the people of the section in which he lived. The desire also to develop new facts in medical science which would extend their blessings to the human race in every land, actuated him at all times.

In the same year that he commenced business on his own account he was married to Miss Martha B. Smith, only child of Col. Wm. R. Smith, of Rome, Ga., one of the bravest, most generous and most enterprising citizens of the State—a pioneer of civilization in the Cherokee portion of Georgia. The daughter inherited the generous nature of her father, the amiability of her mother, and strong common sense from both, fitting her eminently as the life-companion of her worthy and distinguished husband. She has seconded all his endeavors, and by her gentleness and affection has ministered to his happiness all along the passing years, encouraging his efforts at discovery, and rejoicing in every achievement he has made. Fourteen children have distinguished their marriage, nine of whom still live to gladden their hearts and give happiness to their home.

The ambition to perfect himself in his profession was an everactuating influence, causing him in 1855 to enter the Chemical Laboratory of Prof. James C. Booth, of Philadelphia, as a student of analytical chemistry.

In 1856 he graduated at the College of Pharmacy in Philadelphia, and in 1857 received his diploma from Jefferson Medical College, and also from the Obstetrical Institute of Philadelphia.

Thus prepared by assiduous and persistent study and experiments, as well as by the most eminent instruction in America, he offered himself as a practitioner of medicine to the people among whom he lived. So great was his personal popularity and his reputation for thoroughly mastering every subject with which his mind grappled, that his entry upon the practice was a triumph from the beginning. It seemed impossible for him, even at the commencement of his career as a physician, to avoid experiments calculated to render his profession more useful to mankind. Desire for discovery was a part of his nature, and he was ever watchful of opportunities to develop new facts in medical science and new methods of practice. A writer says of him: "In June, 1858, he operated successfully for vesico-vaginal fistula." * *.

In February, 1859, he devised and practiced with success a new method of treating congenital talipes by the use of a curved splint, etc. * *. This method he afterwards extended in its application by the aid of tenotomy, to older children. Encountering a case of special difficulty in vesico-vaginal fistula, in June, 1859, he devised a modification of the methods of Sims and Bozeman, which successfully overcame the obstacles. This method he presented, by invitation, before the Obstetrical Society of London in October, 1859, the fact being reported in the Transactions of the Obstetrical Society of London in 1859, and in the London *Lancet* in 1860 (American reprint for March, 1860).

Prior to his departure for England in 1859, he presided as vice-President of the American Pharmaceutical Association, at its session in Boston, September, 1859.

Accustomed to rely upon himself from early boyhood, he visited Great Britain alone, and made his entry into its principal cities without letters of introduction, and through the medium of his personal celebrity and his earnest, unpretending deportment, gained admission to the most popular medical societies and associations of England, Scotland and Ireland. Prof. Fleetwood Churchill, of Dublin, most cordially received him at his home and presented him to the chief surgeons and obstetricians of that city, through whose influence he was afforded the opportunity to demonstrate his mode of operation for vesico-vaginal fistula. The operation was performed by him on a case in Dublin Hospital, which had been operated upon five times by the surgeon in charge after the method of Sims and Bozeman, and abandoned as incurable. The most eminent surgeons of Scotland and Ireland, who had examined this case, gave their assent to its supposed incurability, and were much surprised and gratified by the success of Dr. Battey. When he left Dublin he was furnished by Dr. Churchill letters of introduction to many eminent surgeons and obstetricians of England, Scotland and Ireland. Wherever he journeyed and sojourned he was treated with extreme cordiality, and was offered various opportunities for superior experiments in surgery and medicine.

From Great Britain he visited Paris, and met with a like cordial reception from the eminent physicans and surgeons of that city, demonstrating his successful methods of surgery on subjects in the hospitals—methods that were then in the advance of the practice of the most distinguished surgeons of the world. Brussels was the next point of his visitation. Thence he returned to America, after a tour remarkable for its cordial receptions by the medical men of progress in the old world, his successful demonstrations of his discoveries, and the high appreciation of his achievements in medical science, destined to bless mankind. He arrived at his home at a most important period in the history of the United States; a period when sectional feeling had been intensified and when the very life of the Republic was seriously involved. He resumed his practice with great gratification, feeling himself much improved by his opportunities in the Old World, and when, finally, the cloud of war began to pour its destructive influence upon his country, as all good and true men, he gave his adhesion and his services to his native section.

He entered the service of the South as medical officer of Colonel Stovall's battalion of artillery, and was soon commissioned surgeon of the 19th Georgia Regiment attached to the Army of Virginia. After the battle of Seven Pines, he was advanced to the position of senior surgeon of Hampton's Brigade, and soon thereafter as senior surgeon of Archer's Brigade, and in this latter position he served with Stonewall Jackson's corps through the memorable campaign of 1862. As the winter came on, he was transferred to Atlanta, Ga., on account of the severity of the climate of Virginia at that season, and was placed in charge of Fair Ground Hospital No. 2. In the following summer he was put in charge of Polk Hospital at Rome, Ga., this transfer being made by order of Surgeon General Moore on account of the extremely delicate health of Mrs. Battey, and the unprotected condition of his family of little children.

In 1864, he performed a successful operation for vesico-vaginal fistula on a lady of high social position, who for twenty-two years had been, on account of this affliction, a recluse. He was assisted in this operation by Surgeon Pim, of the army, but it was performed by his method and resulted in a most gratifying recovery.

He was driven from Rome by the enemy in 1864, leaving his family within their lines, and established himself at the head of hospitals at Atlanta, and at Vineville and Macon, successively, and when Hood made his celebrated advance into Tennessee, Dr. Battey was removed to Lauderdale, Mississippi. In December, 1864, he was stationed in Macon, Ga., opening his hospital in the building of the Academy for the Blind, setting it apart exclusively for treatment of fistula, hernia, etc., and establishing a semi-weekly clinic of operations in these cases, which were attended by many medical officers, who regarded it as of great interest to that branch of the army service. In April, 1865, he was surrendered to the Federal army, and resumed the practice of his profession at his home.

In July, 1866, he performed an operation for complete false anchylosis of the hip-joint, the limb being fixed in a most inconvenient position, entirely disabling the young man from active employment. The ensuing season he was able to make a regular hand with the plow, having recovered altogether.

In May, 1869, he performed his first operation of ovariotomy. The subject was the wife of a physician near Montgomery, Alabama. He removed a dermoid cyst weighing thirty pounds, which contained an abundance of hair, with also teeth and bones. The patient is now living in the enjoyment of excellent health. In July, 1869, he performed the operation of perineal cystotomy for chronic cystitis. The patient recovered. This operation, though previously proposed by eminent surgeons of New York and Philadelphia, had never been successfully performed before.

In August, 1872, he performed an operation by which premature change of life was effected in a young lady by the extirpation of the ovaries, thus arresting promptly certain vascular and nervous disorders threatening her life. This operation, which was entirely without precedent, and original in conception, also was performed with eminent success. In 1873 he made an able and successful defense of this operation before the Medical Association of Georgia, and in 1877 a triumphant defense before the American Gynæcological Society at its session in Boston. A report of it was first published to the world in THE ATLANTA MEDICAL AND SURGICAL JOURNAL for September, 1872.

It was in March, 1873, that he made the original discovery that water could be injected *per anum*, by means of an ordinary syringe, in a living subject, and carried through the entire canal into the stomach, thence out at the mouth, notwithstanding the ileo-cæcal and pyloric valves, previously supposed to offer insuperable obstacles. He has repeated this operation in cases of strangulated hernia after its reduction *en masse*. He demonstrated this discovery on the cadaver at the Atlanta Medical College in Jan., 1874. It has subsequently been frequently practiced by others as well as himself.

In 1873 he became connected as corresponding editor, and then as editor-in-chief of this journal. The same year he was called to, and accepted the Chair of Obstetrics in the Atlanta Medical College. In this position he continued for two years.

The operation of vaginal ovariotomy was successfully performed by him in April, 1874, it being the third case of its successful performance on record. In November, 1876, he removed a fibro-cystic growth weighing four and half pounds from the submaxillary and carotid space, and a precisely similar growth of one and a half pounds weight was, two months thereafter, taken by him from the neck of another man. In both cases he was successful.

He introduced to the profession in 1877, through *The American Practitioner*, a new uterine escharotic and alterative which he designated as iodized phenol. This has been well received and is regarded as an important addition to the resources of the profession in uterine therapeutics. It is, in many cases, considered superior to nitrate of silver, sulphate of zinc, nitric acid and other topical applications. He was elected President of the Georgia Medical Association in April, 1876. He was at one time a member of the Judicial Council of the American Medical Association, and is one of the founders and fellows of the American Gynæcological Society.

The late Dr. J. Marion Sims thus writes of Dr. Battey and his operation in the *British Medical Journal* of December, 1877:

"When Battey reasoned out his operation of extirpating the ovaries to effect change of life, he reasoned out a truism, for the removal of the ovaries must necessarily stop ovulation, which constitutes a *de facto* change of life, whether the menses recur afterwards or not. The cessation of menstruation may then be regarded as the sign of change of life, but not the actual change. From this point of view, Battey was right in theory; and experience shows that he is right in practice. His operation of extirpating the ovaries to arrest the menstrual molimen is based upon sound physiological doctrine; and in practice it accomplishes what it proposes to do. For we find that, when both ovaries are neatly and cleanly removed, the menstrual molimen ceases. But, if they be imperfectly removed, the menstrual molimen recurs as regularly as it did before the operation.

The term normal ovariotomy, applied by Battey to his operation, is a misnomer; for, in all cases requiring operation, the ovaries are never found in a normal state. This term has been much and justly criticised; and Battey asked me some time ago to give his operation a name. I would like to see it recognized by the profession as "Battey's Operation." I think he is fully entitled to that honor. He was the first to grasp, in its widest range, the influence and effects upon the general system of what he calls an "unrelieved menstrual molimen." He was the first to suggest a method of cure; he was the first to carry out his own suggestion, and to perform an operation for the cure of a disease that had never been cured before. He performed the operation on his own responsibility, with no great authority to sustain him. He demonstrated the correctness of the principles upon which his operation was based, by proving its success in practice. He established a precedent that may now be followed with safety, and opened up a new field of research that promises results as grand as those now achieved by ovariotomy. He has in many instances raised sorrowing and hopelessly incurable women from a perfect slough of despair, from indescribable suffering, from epileptic convulsions, from threatened insanity, and in some instances from impending and certain death, and restored them to health. . . . . The difficulty already encountered in finding a name sufficiently distinctive and characteristic of this operation justifies us in calling it Battey's Operation. I cherish the hope that the profession in Europe will unite with us in America in giving it the name of the man who originated the operation, and who has, by the most indomitable courage, succeeded in proving its usefulness. He has won the honor, and let him wear it."

In June, 1882, in order to supply better facilities for the performance of his operation, which had proved such a signal blessing to suffering humanity, and made him famous as a surgeon and philanthropist, he established at the city of Rome an infirmary, supplied with all the necessary means for the care and comfort of patients and the success of his practice. The wisdom of this is seen in the fact that since the opening of this infirmary he has lost only one case out of thirty-nine operations. This is a record that any surgeon might well be proud of.

## Figures and Tables

**Figure f1:**